# Autophagy as an emerging therapy target for ovarian carcinoma

**DOI:** 10.18632/oncotarget.13080

**Published:** 2016-11-04

**Authors:** Lei Zhan, Yu Zhang, Wenyan Wang, Enxue Song, Yijun Fan, Jun Li, Bing Wei

**Affiliations:** ^1^ Department of gynecology and obstetrics, The Second Affiliated Hospital of Anhui Medical University, Hefei 230601, China; ^2^ School of Pharmacy, Anhui Key Laboratory of Bioactivity of Natural Products, Anhui Medical University, Hefei 230032, China

**Keywords:** autophagy, dual functions, ovarian carcinoma, mechanism, therapies

## Abstract

Autophagy is a conserved cellular self-digestion pathway for maintenance of homeostasis under basal and stressed conditions. Autophagy plays pivotal roles in the pathogenesis of many diseases, such as aging-related diseases, autoimmune diseases, cardiovascular diseases, and cancers. Of special note is that accumulating data suggest an intimate relationship between autophagy and ovarian carcinoma. Autophagy is well identified to act as either as a tumor-suppressor or as a tumor-promoter in ovarian carcinoma. The exact function of autophagy in ovarian carcinoma is highly dependent on the circumstances of cancer including hypoxic, nutrient-deficient, chemotherapy and so on. However, the mechanism underlying autophagy associated with ovarian carcinoma remains elusive, the precise role of autophagy in ovarian carcinoma also remains undetermined. In this review, we tried to sum up and discuss recent research achievements of autophagy in ovarian cancer. Moreover, waves of novel therapies ways for ovarian carcinoma based on the functions of autophagy were collected.

## INTRODUCTION

Ovarian carcinoma is the fifth leading cause of death from all gynecological malignancies in development countries. Despite the improvement of surgical techniques and the advent of more effective therapeutics, the overall 5-year survival rate of ovarian cancer is approximately 30-40% [1, 2]. Therefore, it is necessary to explore novel avenues to protect against ovarian cancer [3–5]. In the past few decades, a growing body of researches have been illustrated an intimate correlation between autophagy and ovarian carcinoma which may provide new treatment option for ovarian carcinaoma [6].

**Figure 1:**
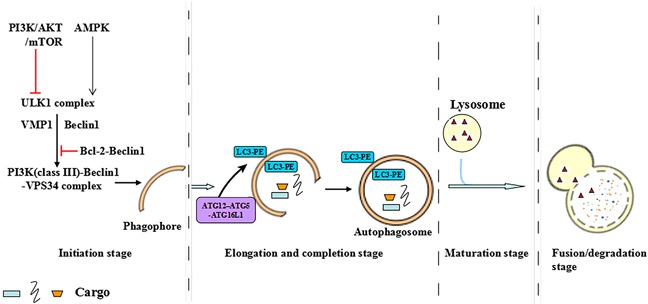
The multistep processes of autophagy The multistep process of autophagy is controlled by the autophagy-related ATG groups and occurs in four steps: initiation stage, elongation and completion stage, maturation stage and fusion or degradation stage.

Autophagy is an evolutionarily conserved cellular self-digestion pathway for maintenance of homeostasis by recycling lysosome-dependent intracellular soluble macromolecules, organelles and microorganisms [7, 8]. It is well known that there are three different forms of autophagy which are macroautophagy, chaperone-mediated autophagy and microautophagy respectively [9]. Macroautophagy refers to simply as autophagy and this review focuses on macroautophagy [9]. Autophagy exists at basal level in all cell types and could be dramatically activated by a wide diversity of stresses, such as starvation, infection, hormones, oncogenes, oxidative stress and endoplasmic reticulum (ER) stress [10, 11]. Furthermore, the stresses-activated autophagy is correlated with many key signal transduction pathways, including phosphatidylinositol-3-kinase (PI3K), myostatin, proteasome, autophagy-lysosome pathways and so on [12]. The principal autophagic signaling that have been implicated in autophagy progression are the PI3K/AKT/mammalian target of rapamycin (mTOR) pathway and liver kinase B1 (LKB1)/adenosine monophosphate-activated protein kinase (AMPK) pathway. PI3K/AKT/mTOR activation inhibits autophagy progression, whereas LKB1/AMPK activation contributes to autophagy progression [13]. The multistep processes of autophagy is controlled by the autophagy-related ATG groups and occurs in four steps as follows [13, 14]: (i) Initiation stage, the activation of ULK1 complex (ULK1, ULK2, ATG13, FIP200, and ATG101) translocates to the ER and transiently associates with VMP1 and Beclin1 in the initiation stage, further facilitating the activation of the ER-localized autophagy-specific class III phosphatidylinositol-3-OH kinase (PI3K)-Beclin1-VPS34 complex. PI3K-Beclin1-VPS34 complex then recruits to the mitochondria, ER and golgi apparatus to form an isolated membrane phagophore through recycling endosome or plasma membrane. (ii) Elongation and completion stage, in this stage, cargo recognition and elongation of phagophore contribute to the formation of double-membrane structure, autophagosome. It is noted that the formation of double-membrane structure autophagosomes is the landmark event in autophagy and which requires two ubiquitin-like protein conjugation systems: the first is the ATG7 and ATG10 enzymes produced ATG12-ATG5-ATG16 conjugation system. The other conjugate system is the phosphatidylethanolamine (PE)-LC3. LC3, which consists two forms the cytoplasmic form LC3-I and the processed form LC3-II, is synthesized as an inactive form called pro-LC3. This form must be cleaved by a protease of the ATG4 family of cysteine proteinases to render it capable of undergoing a series of reactions first with ATG7 and then with ATG3, which finally end in the covalent binding of LC3 to a molecule of PE. Once autophagosome has ended its life cycle, the remaining LC3 molecules attached to the outer leaflet of the autolysosomal membrane are cleaved again by a protease of the ATG4 family of cysteine proteinases to allow its re-use by the autophagic cellular machinery. (iii) Maturation stage, the cargo sequestration is completed and the outer membrane of the autophagosome fuses with the lysosome to become an autolysosome, which leading to the degradation of autophagosomal contents by lysosomal enzymes. (iv) Fusion or degradation stage, the cargo is broken down and released into the cytosol in this stage (Figure 1).

Autophagy earns wide attentions owing to its intimate correlation in a multitude of diseases and its potential as a target in diseases therapy [15, 16]. It is worth mentioning in advance that autophagy play either cytotoxic function or cytoprotective function depending on the condition of diseases. In general, decreased levels of autophagy contribute to cell proliferation. Whereas increased levels of autophagy can also promote cell survival in the context of hypoxic, nutrient-deficient [17]. On the basis of that autophagy was revealed to act either as a tumor-suppressor or as a tumor-promoter. For example, decreased levels of autophagy promoted cancer cell proliferation in the early stages of tumorigenesis, stimulating autophagy could be beneficial for cancer prevention [18, 19]. Alternatively, autophagy induction promoted cancer cell survival in response to hypoxic and nutrient-deficient tumor microenvironments, deletion of autophagy genes resulted in tumor cell death in hypoxic [20, 21].

Noteworthily, rapidly accumulating attention is being paid to the role of autophagy in ovarian carcinoma and its potential as a promising therapeutic target [22, 23]. The aim of this review is to sum up the collective knowledge of autophagy in ovarian cancer and discuss the therapies target for ovarian cancer based on the functions of autophagy.

## AUTOPHAGY AND OVARIAN CARCINOMA

### Autophagy-related proteins and ovarian carcinoma

During autophagy induction, LC3-I transforms to LC3-II and forms autophagosome in the outer and inner membrane, implying that LC3-II expression represents autophagy level [24]. It was indicated that LC3 was expressed at a lower level in ovarian cancer tissues as compared to benign and borderline ovarian tumors. Furthermore, the expression of LC3 was lower in FIGO stages III and IV than in stages I and II [25]. A diversity of studies implied that promoting autophagy-related cell death was required for up-regulation of LC3-II levels in ovarian cancer tissues and cells [26–28].

Autophagy process was involved in down-regulation of autophagy-related proteinp62 [29]. p62 binds to ubiquitinated protein aggregates and transports them to the autophagosomes in the late stage of autophagy [30]. For example, it was indicated that p62 bond directly to LC3 to facilitate degradation of ubiquitinated protein aggregates [31]. High expression of cytoplasm p62 was found in ovarian cancer tissues and cells [28, 29]. Furthermore, high expression of cytoplasmic p62 in ovarian cancer was positive correlated with serous carcinoma, advanced stage, presence of residual tumor and low overall survival rate, which strongly suggesting that high expression of cytoplasmic p62 was a prognostic biomarker in ovarian cancer [32]. Down-regulating p62 contributed to autophagic-and caspase-mediated cell death in a p53-independent manner in chemoresistant ovarian cancer cell lines [33].

As stated above, Beclin1 was involved in autophagosome formation through assembling PI3K-Beclin1-VPS34 complex, Bcl-2 inhibited autophagy through binding to Beclin1. It was interesting to detect that 40-75% of sporadic ovarian cancers exhibited a monoallelic deletion of the gene that encodes Beclin1 [34]. The expression of Beclin1 was dramatically higher in the samples of benign and borderline ovarian tumors than those in malignant epithelial ovarian cancers, higher levels of Beclin1 were negative associated with advanced FIGO stage and histological grade [25]. Notably, patients with high Beclin1 levels exhibited better survival than those with low Beclin1 levels [35]. Inhibition of autophagy contributed to ovarian carcinoma development was required for suppression of Beclin1 and up-regulation of Bcl-2 [36]. Consistently, in ovarian carcinoma cohort, decreased expression of Beclin1 was inversely correlated with Bcl-xL, which was an antiapoptotic protein from the Bcl-2 family. Low Beclin1/high Bcl-xL ovarian carcinoma group exhibited the lowest survival rate [37]. It was worth nothing that the tumor suppressive effects of Beclin1 were witnessed to be dispensable for autophagy induction because efficient siRNA-mediated Beclin1 knockdown did not attenuate autophagy induction in ovarian tumor cells [38, 39].

ATG groups were also expressed at a lower level in ovarian cancer tissues and cells. ATG5-ATG12 conjugates were essential to LC3-I to LC3-II conversion. Studies convincingly showed that the expressions of ATG7-acivated ATG5-ATG12 conjugates were consistent with LC3-II and Beclin1 in ovarian cancer [36, 39, 40]. Unlike the Beclin1, efficient ATG5 and ATG7 knockdown could block autophagy induction in ovarian cancer cell lines, as evidenced by suppression of LC3-II [39].

Based on these above findings, it is reasonable to assume that down-regulation of autophagy appears to be prominent in the development of ovarian cancer. Inconsistently, the study of Dai and colleagues described an up-regulation of ATG9A expression in ovarian carcinomas. Furthermore, the percentage of positive ATG9A expression was significantly higher in cases with clinical stage III and IV ovarian cancer than in cases with clinical stage II ovarian cancer, elevated ATG9A was an independent poor prognostic predictor and negative related with overall survival and progression-free survival in ovarian cancer [41].

### Autophagy-related signaling pathways in ovarian carcinoma

The induction of autophagy was intimately involved in apoptosis promotion and proliferation inhibition in ovarian carcinoma. Therefore, the signaling pathways which have essential roles in proliferation or apoptosis were correlated with ovarian carcinoma development by regulating autophagy. For example, it was demonstrated that inhibition of the PI3K/Akt/mTOR and Ras/MAP signaling pathways could restrict ovarian cancer development by activating autophagy [3, 42]. Zi et al. also detected that danusertib induced cell apoptosis and autophagy in ovarian carcinoma was involved in PI3K/Akt/mTOR signaling pathway inhibition [43]. Furthermore, Bahrami and colleagues indicated that monepantel induced autophagy in human ovarian cancer cells through disruption of the mTOR/p70S6K signalling pathway [44]. In contrast, activation of p38 MAPK and JNK signaling pathways contributed to the induction of autophagy in ovarian cancer cells [45–47]. For instance, Neferine induced autophagy of human ovarian cancer cells *via* p38 MAPK/JNK activation [47]. Inhibition of JNK3 could promote apoptosis and autophagy in chemoresistant human ovarian cancer cells [45].

### Chemotherapy resistance with autophagy in ovarian carcinoma

Cisplatin is a first-line therapeutic agent used in therapy of ovarian carcinoma. However, cisplatin resistance always exists in ovarian cancer patients and resulted in treatment failure [48]. It was suggested that autophagy had a protective mechanisms in the regulation of chemosensitivity to cisplatin with virtually promotion of autophagy contributed to cisplatin resistance in human ovarian cancer, inhibition of autophagy through using autophagy inhibitor and Beclin1 small interfering RNA (SiRNA) increased cisplatin-induced cell death and apoptosis [49, 50]. Consistently, Bao and colleagues found NF-E2-related factor 2 (Nrf2) contributed to cisplatin resistance through activating autophagy in ovarian carcinoma [48]. Furthermore, methionine synthase reductase (MTRR), which was a promoter of carcinogenesis, was found to be increased in ovarian carcinoma. Inhibiting MTRR expression suppressed cisplatin resistance by reducing autophagy [51]. A recent study by Xiao et al. also suggested that the oncoprotein, YAP, induced cisplatin resistance through activating autophagy in human ovarian carcinoma cells [52]. These results indicated that autophagy acted as a protector in cisplatin treatment ovarian carcinoma. The mechanism underlying autophagy contributed to cisplatin resistance was also studied. Yin et al. showed that phosphatase 2A catalytic subunit (PP2Ac), which was a target for cisplatin, suppressed the accumulation of LC3-II and restored p62, knocking down of PP2A promoted autophagy in cisplatin-resistant ovarian cancer cells, suggesting protective autophagy inhibited by PP2Ac is a part of the mechanism to how certain ovarian cancers are resistant to cisplatin [53]. Furthermore, a novel mechanism was estimated by Wang et al., they showed that cisplatin activated ERK and subsequently promoted ERK induced-autophagy, which counteracted cisplatin-induced cell death. Knockdown of ERK decreased cisplatin-induced autophagy and increased cisplatin-induced cell death [54]. Paclitaxel is also identified as a first-line chemotherapeutic agent against ovarian cancer, whereas the chemotherapy resistant still exists [55]. Zhang et al. revealed autophagy as a promoter in paclitaxel resistant in ovarian cancer. The up-regulation of autophagy which was induced by thioredoxin domain containing 17 (TXNDC17) promoted paclitaxel resistance in ovarian cancer [56].

Inconsistently, Khurana and colleagues identified that p62 levels were elevated in chemoresistant ovarian cancer cell lines compared to chemosensitive ovarian cancer cell lines, which suggested autophagy was down-regulated in chemoresistant ovarian cancer cell lines [33].

### Tumor suppressor genes with autophagy in ovarian carcinoma

Aplasia Ras homolog member I (ARHI; DIRAS3) is an imprinted tumor suppressor gene that is responsible for initiating programmed cell death and inhibiting cancer cell growth, and ARHI is down-regulated in 60% of ovarian cancers [57]. Over-expression of ARHI led to an accelerated level of autophagy by suppressing PI3K/AKT and reducing the expression of Bcl-2 in ovarian cancer cells [57]. Consistently, Lu and colleagues suggested that ARHI promoted autophagosome biogenesis and triggers vesicle nucleation by binding to Beclin1, disrupting Beclin1-Bcl-2 interaction and promoting assembly of the Beclin1-PIK3C3-ATG14 initiation complex. Furthermore, ARHI-Beclin1 interactions were strengthened and autophagy facilitated the survival of nutrient-deprived dormant ovarian cancer cells [58]. Recently, they further clearly illustrated that in ovarian cancer cells, ARHI contributed to the induction of autophagy by down-regulating the epidermal growth factor receptor, inhibiting PI3K and Ras/MAPK signaling and activating the FOXo3a-mediated induction of Rab7 [42]. More recently, Lu et al. found ARHI induced autophagy could enhance sensitivity to cisplatin in ovarian cancer cell lines and xenografts [59], which was inconsistent with that up-regulation of autophagy always contributed to cisplatin resistance in human ovarian cancer [49, 50].

p53 is a well known tumor suppressor protein that was intimately correlated with autophagy, recent reports have indicated that autophagy can be regulated by p53 either for a protective or destructive effect which depending on the subcellular localization. Nuclear p53 facilitated autophagy, whereas cytoplasmic p53 mainly inhibited autophagy and low cytosolic levels of p53 could trigger autophagy [15, 60, 61]. It has been highlighted that p53 was mutated in >96% of high-grade serous ovarian carcinomas, mutant p53 contributed to ovarian cancer by promoting tumor differentiation, metastasis, and responsiveness to steroid hormones [62, 63]. Despite a substantial body of evidence indicated the close relationship between p53 and autophagy, researches for their connection in ovarian cancer were limited. Kong and colleagues showed that wild-type p53 sensitized multidrug resistant (MDR) human ovarian carcinoma cell lines to vincristine, cisplatin, pirarubicin and etoposide by inducing apoptosis and decreasing autophagy, while mutant p53 reversed the MDR by trigging autophagic cell death, necrosis and apoptosis [64].

The phosphatase and tensin homolog (PTEN) protein is one of the most commonly mutated tumor suppressor genes with dual specificity phosphatase activity [65]. PTEN is able to initiate autophagy by dephosphorylating PIP3 in the PI3K/AKT/mTOR signaling pathway, loss of PTEN in cancers leads to decreased levels of autophagy and facilitates tumorigenesis [66, 67]. Studies found mutations of PTEN commonly occur in ovarian cancer [63, 68]. Study also suggested that low level of Beclin-1 and PTEN resulted in drug resistance through reducing autophagic activity in the epithelial ovarian cancer tissues [69].

### miRNAs with autophagy in ovarian carcinoma

miRNAs are known as one of the most popular epigenetic genes, are a class of small noncoding RNAs of about 22 nucleotides in length. miRNAs inhibit gene expression transcriptionally and post-transcriptionally [70]. It has been well recognized that a vast majority of miRNAs deregulation extensively participated in the progression of cancers by regulating autophagy especially the ATGs proteins [71]. In ovarian cancer, the oncomiR miR-30d was illustrated to impair autophagy by suppressing a multitude of autophagy-related genes including Beclin1, BNIP3L, ATG12, ATG5, ATG2 directly and inhibiting LC3-I conversion to LC3-II [72]. Moreover, transfection of miR-29b which was tumor suppressor could inhibit ATG9A mRNA expression directly in ovarian cancer [41]. In another study by Jiang et al., cisplatin-resistance resulted in miR-152 down-regulation and autophagy up-regualtion in ovarian cancer, over-expression of miR-152 sensitized ovarian cancer cells to cisplatin-induced apoptosis by inhibiting ATG14 expression and autophagy-induced cyto-protection [73]. In addition to the above evidence that miRNAs participate in the progression of ovarian cancer by correlating with autophay, there are a wide range of potential connections between miRNAs and autuphagy in ovarian cancer, which need further determined in the future [71].

### Induction of autophagy as a therapeutic option

As we talked above that high level of autophagy contributed to cell apoptosis generally, whereupon, the therapeutic relevance of induction of autophagy as an option for ovarian cancer therapy is widely researched. The following numerous studies conform to this opinion. Dasatinib and dihydroptychantol 2 were showed to inhibit ovarian cancer cells (SKOV3 and Hey) growth by inducing the expression of LC3B-II, Beclin1 and Atg12 [74, 75]. In A2780 ovarian cancer cells, C2-ceramide treatment benefited to cells apoptosis was through up-regulation of LC3-II [76]. MORAB-003 (farletuzumab), which is a humanized anti-folate receptor alpha (FRα) monoclonal antibody derived from optimization of the LK26 molecule, contributed to human ovarian cancer cells (A2780, HeyA8, SKOV3ip1 and IGROV1) death associated with increased expression of LC3-II and enriched autophagic vacuolization in ovarian cancer [74]. Furthermore, cotreatment of suberoylanilide hydroxamic acid (SAHA) and PP242 inhibited growth of ovarian cancer cells (SKOV3 and A2780) synergistically by promoting LC3-II expression and decreasing p62 expression [77]. Likewise, combination treatment of a PARP inhibitor and a selective EGFR inhibitor erlotinib in ovarian cancer A2780 xenografts had an apparently enhanced antitumor effect *via* enhancing autophagy compared to their monotherapy [78]. The result also existed in the combination treatment of saracatinib and fulvestrant in patients with ovarian cancer [79]. Furthermore, study suggested that resveratrol could induce human ovarian cancer cells (OVCAR-3 and Caov-3) apoptosis by triggering ATG5 expression and promoting LC3 cleavage, inhibiting autophagy with chloroquine reduced resveratrol-induced cells death [80]. Radiotherapy has been offered in the palliative setting after first-line chemotherapy failure for ovarian cancer [81]. It was estimated that treatment ovarian cancer with radiation was implicated with the activation of autophagy. A recent *in vivo* study investigated that andrographolide acted as a strong radiosensitizer in human ovarian SKOV3 xenografts by increasing the level of Beclin1 and ATG5 and the conversion from LC3-I to LC3-II [82]. In another ovarian cancer cell, studies convincingly illustrated that ghrelin, emodin and B19, which was a novel monocarbonyl analogue of curcumin, played an antitumor effect of on HO-8910 cells by inducing autophagy, including increased level of MAP LC3, Beclin1, Atg12-Atg5 complex, promotion of transformation of LC3-I to LC3-II and decreased level of p62 [83–85]. In a recent *in vitro* and *in vivo* study in chemoresistant ovarain cancer cell lines, quinacrine was observed to promote autophagosome accumulation and enhance autophagic flux by clearance of p62, knockdown of p62 facilitated quinacrine-activated cell death and chemosensitization [33]. Of special note is that, Mono-Pt, which is a novel monofunctional platinum (II) complex, played an anticancer effect in ovarian cancer *via* increasing the punctate distribution of LC3 and the ratio of LC3-II to LC3-I, Mono-Pt-induced cell death was significantly restricted by the knockdown of either Beclin1, ATG7 or the autophagy inhibitors 3-methyladenine, chloroquine and bafilomycin A1. The charm of these results was distinct from the cisplatin, which triggered autophagy to act as a protector in ovarian cancer [23]. It was interesting to notice that vaccinia virus induced ovarian cancer cells programmed necrosis by changing the rate of lysosomal degradation of LC3B-II, but did not increase autophagic flux and did not rely on autophagy-induced death [75]. Noteworthily, BH3 mimetic S1 led to autophagy activation that attenuated ER stress-induced apoptosis in SKOV3/DDP cells, inhibition of autophagy increased S1-induced ER stress-stimulated apoptosis at early time points (2 and 4 h). However, the activation of autophagy was attenuated and S1-induced ER stress-stimulated apoptosis acted as a promoter in killing SKOV3/DDP cells in the end [86].

It has been well studied the therapeutic mechanism under which induced autophagy in ovarian cancer treatment. Studies extensively showed that the induction of autophagy was involved in inhibition of AKT/mTOR/ (p70S6K) signaling pathway [35, 38, 65, 74, 76, 78, 79] and activation of MAPK (ERK), JNK, and AMPK signaling pathways [36, 38, 76, 87]. Inhibition of AKT/mTOR/(p70S6K) signaling pathway and activation of MAPK (ERK), JNK and AMPK signaling pathways increased autophagy-induced cell apoptosis in ovarian cancer. Furthermore, drug-induced autophagy induction was also implicated in reduction of Bcl-2, over-expression of Bcl-2 inhibited autophagy-induced apoptosis [75, 88]. Jin et al. demonstrated C2-ceramide-induced autophagy in A2780 ovarian cancer cells was though activating forkhead box O3 (FOXO3) and its target genes [76]. In addition, G129R induced the accumulation of autophagy by blocking the tumoral PRL/PRLR axis in ovarian cancer [28].

### Inhibition of autophagy as a therapeutic option

As we stated above, autophagy can act as a survival promoter in the context of hypoxic, nutrient-deficient environments and chemotherapy. It has been well elucidated that autophagy acted as a protector in the chemotherapy of ovarian cancer. For example, EGFR inhibitor AG1478 and metformin-induced ovarian cancer cells apoptosis resulted in up-regulation of autophagy characterized by elevation of LC3-II, Beclin1, ATG5-ATG12 complex and decreased p62, inhibition of autophagy significantly facilitated the anticancer effect of AG1478 and metformin [89, 90]. Cisplatin-resistant in ovarian cancer was involved in up-regulation of autophagy. Suppression of autophagy in cisplatin-resistant ovarian cancer cells contributed to the therapeutic effect of cisplatin. Studies found that dihydroartemisinin, bortezomib and MTRR silencing inhibited cisplatin-resistant and potentiated the anticancer effect of cisplatin *via* inhibiting autophagy in ovarian cancer cells [43, 81, 82]. In line therewith, Chung et al. identified that ellagic acid was able to assist the chemotherapy efficacy of doxorubicin by inhibiting autophagy in ovarian carcinoma ES-2 and PA-1 cells [91]. A recent study showed the effectiveness of elaiophylin, a novel autophagy inhibitor, in the treatment of ovarian cancer cells and found that elaiophylin decreased cell viability in combination with cisplatin or under hypoxia conditions [92]. In the ovarian cancer xenograft mouse model, endometrioid SKOV-3 cells were shown to be resistant to 4-(N-(S-penicillaminylacetyl) amino) phenylarsonous acid (PENAO) due to an ability to cope with PENAO-induced oxidative stress. Whereas using mTORC1 inhibitor in combination with PENAO synergistically to inhibit SKOV-3 cells proliferation [93]. Lately, Wang et al. implicated that autophagy could protect ovarian cancer-associated fibroblasts against oxidative stress, blockage of autophagy could sensitize ovarian cancer associated fibroblasts to chemotherapeutic drug cisplatin [94].

The above findings suggested that chemotherapy together with inhibition of autophagy may be the alternative way to treat ovarian cancer [27, 95, 96]. Furthermore, inhibition of autophagy was explicitly observed to convert the combination of FTY720 with cisplatin from an antagonistic into an additive effect toward killing ovarian cancer cells [97]. Inconsistently, Ali et al. suggested that dorsomorphin successfully resensitized drug-resistant ovarian cancer cells to the killing effects of platinum agents by inducing autophagy [98]. It was worth nothing that simvastatin was postulated to activate and block the autophagy pathway at different points in the treatment for ovarian cancer [99].

## CONCLUSION AND PERSPECTIVES

**Figure 2:**
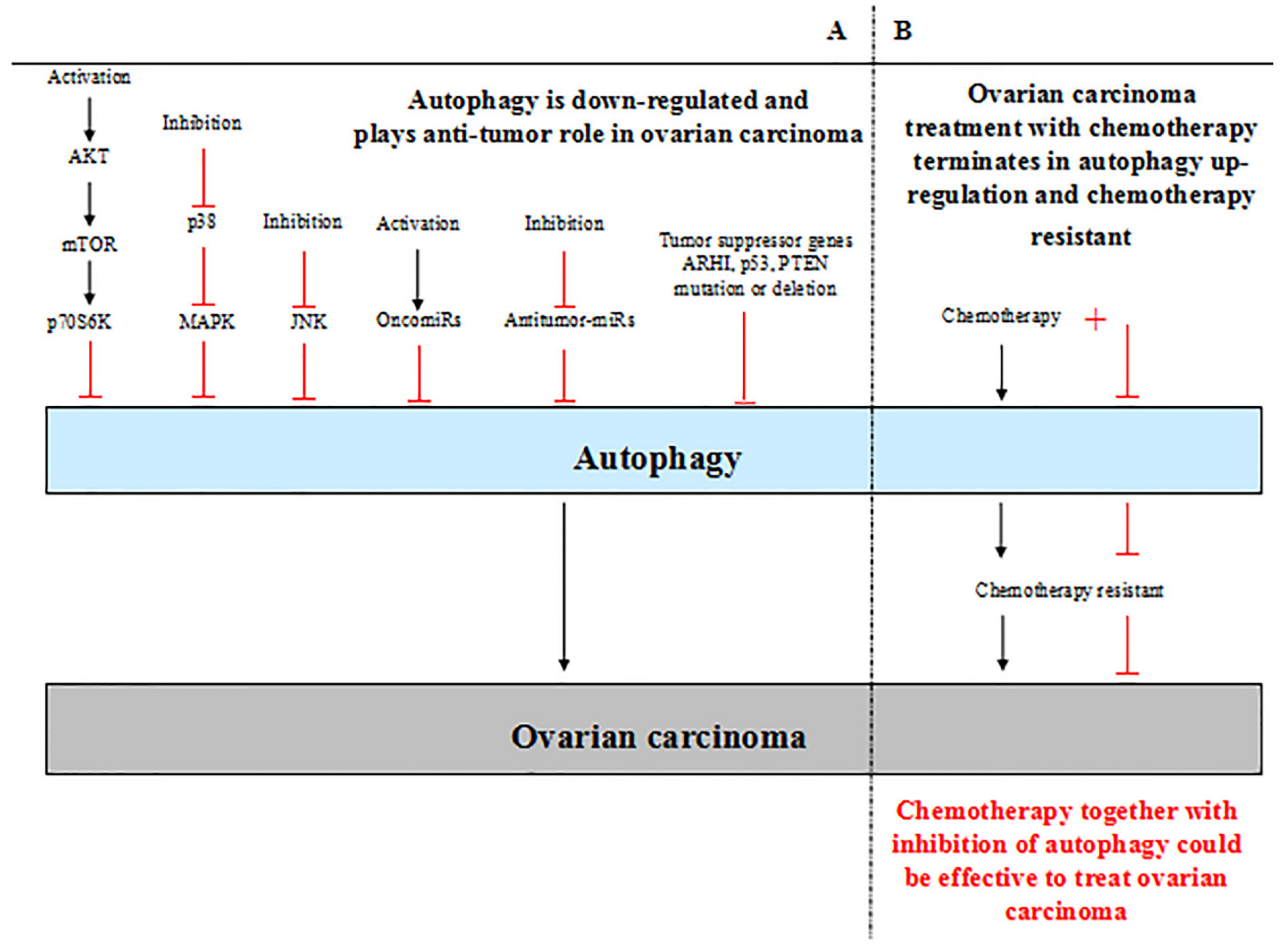
**A.** Autophagy plays dual roles in ovarian carcinoma. The level of autophagy is down-regulated in ovarian carcinoma generally. In such context, autophagy plays an anti-tumor role in ovarian carcinoma; low level of autophagy contributes to ovarian carcinoma develpoment. The activation of AKT/mTOR signaling pathway and inhibition of p38 MAPK and JNK signaling pathways lead to low level of autophagy in ovarian carcinoma. Furthermore, activation of oncomiRs inhibit autophagy directly, inhibition of antitumor-miRs also lead to down-regulation of autophagy. In addition, the tumor suppressor genes mutation or deletion causes inhibition of autophagy in ovarian carcinoma, such as ARHI, p53, PTEN. **B.** In the context of ovarian carcinoma treatment with chemotherapy, autophagy is up-regulated and terminates in chemotherapy resistant. Chemotherapy together with inhibition of autophagy could inhibit chemotherapy resistant and be effective to treat ovarian carcinoma.

By absorbing and integrating the previous studies, the most established conclusion is that autophagy plays dual role in ovarian carcinoma (Figure 2). In general, the level of autophagy is down-regulated in ovarian carcinoma. In such context, autophagy acts a protector in ovarian carcinoma, promoting autophagy contributes to inhibition of ovarian carcinoma development. The mechanism underlying autophagy in the progression of ovarian carcinoma involves in autophagy-related signaling pathways activation or inhibition, autophagy-related tumor suppressor genes mutation or deletion and abnormal miRNAs expression (Table 1). However, in the context of ovarian carcinoma treatment with chemotherapy, autophagy is up-regulated and tends to restrict the function of chemotherapy, which terminates in chemotherapy resistant. So when the treatment is replaced by chemotherapy together with inhibition of autophagy, which will be more effective. Nevertheless, the perspective is controversial; and the complicated behavior of autophagy in ovarian carcinoma makes it frustrated to treat ovarian carcinoma by promoting or inhibiting autophagy simply. Moreover, the precise mechanism of autophagy in ovarian carcinoma also remains to be determined.

**Table 1:** Autophagy-related genes in ovarian carcinom

Gene	Expression	Function	References
LC3-II	Down	Promoting autophagy-related cell death is required for up-regulation of LC3-II levels in ovarian cancer tissues and cells.	[25–28]
p62	Up	High expression of cytoplasmic p62 in ovarian cancer is positive correlated with serous carcinoma, advanced stage, presence of residual tumor and low overall survival rate.	[32]
Beclin1	Down	High levels of Beclin1 are negative associated with advanced FIGO stage, histological grade, and exhibit better survival. Down-regulation of Beclin1 contributes to ovarian carcinoma development is through up-regulating Bcl-xL.	[25, 35–37]
ATG5, ATG7	Down	Down-regulation of ATG5 and ATG7 block autophagy induction in ovarian cancer cell lines.	[39]
ATG9A	Up	Elevated ATG9A is negative related with overall survival and progression-free survival in ovarian cancer.	[41]
PI3K/Akt/mTOR	Activation	Activation of PI3K/Akt/mTOR signaling pathway promotes ovarian cancer development by inhibiting autophagy.	[3, 42, 43]
Ras/MAP	Activation	Activation of Ras/MAP signaling pathway promotes ovarian cancer development by inhibiting autophagy.	[42]
p38 MAPK/JNK	Suppression	Suppression of p38 MAPK/JNK signaling pathway contributes to ovarian cancer development by inhibiting autophagy.	[45–47]
ARHI (DIRAS3)	Down	Down-regulated ARHI accelerates the development of ovarian cancer by restraining autophagy.	[57]
p53	Down	Wild-type p53 sensitizes multidrug resistant human ovarian carcinoma cell lines to chemotherapy by decreasing autophagy, mutant p53 reverses the multidrug resistant by trigging autophagic cell death, necrosis and apoptosis.	[64]
PTEN	Down	Low level of PTEN resulted in drug resistance through reducing autophagic activity in the epithelial ovarian cancer tissues.	[69]
miR-30d	Up	Up-regulation of miR-30d contributes to ovarian cancer development by suppressing Beclin1, BNIP3L, ATG12, ATG5, ATG2 directly and inhibiting LC3-I conversion to LC3-II.	[72]
miR-29b	Down	Transfection of miR-29b inhibits ovarian cancer development by suppressing ATG9A mRNA expression directly.	[41]
miR-152	Down	Over-expression of miR-152 sensitizes ovarian cancer cells to cisplatin-induced apoptosis by inhibiting ATG14 expression and autophagy-induced cyto-protection.	[73]

Although numerous of evidence indicates autophagy modulation could be a potential therapeutic method for ovarian carcinoma, the clinical application is limited. Hopefully, as researches continued, we may finally have the opportunity to discover the clear picture of autophagy in ovarian carcinoma and identify potential therapeutic strategy approach for ovarian carcinoma by regulating autophagy in clinical.
